# The oncolytic peptide LTX-315 triggers immunogenic cell death

**DOI:** 10.1038/cddis.2016.47

**Published:** 2016-03-10

**Authors:** H Zhou, S Forveille, A Sauvat, T Yamazaki, L Senovilla, Y Ma, P Liu, H Yang, L Bezu, K Müller, L Zitvogel, Ø Rekdal, O Kepp, G Kroemer

**Affiliations:** 1 Metabolomics and Cell Biology Platforms, Gustave Roussy Comprehensive Cancer Institute, Villejuif, France; 2 Equipe 11 labellisée Ligue contre le Cancer, Centre de Recherche des Cordeliers, INSERM U1138, 15 rue de l'Ecole de Médecine, Paris, France; 3 Sorbonne Paris Cité, Université Paris Descartes, 15 rue de l'Ecole de Médecine, Paris, France; 4 University of Paris Sud XI, Kremlin Bicêtre, France; 5 Université Pierre et Marie Curie, 15 rue de l'Ecole de Médecine, Paris, France; 6 Department of Immuno-Oncology, Institut de Cancérologie, Gustave Roussy Cancer Campus, 114 rue Edouard Vaillant, Villejuif, France; 7 Institut National de la Santé et de la Recherche Medicale (INSERM), U1015, Villejuif, France; 8 Center of Clinical Investigations in Biotherapies of Cancer (CICBT) 507, Villejuif, France; 9 Gustave Roussy Comprehensive Cancer Center, Villejuif, France; 10 CNRS, UMR8122, Villejuif, France; 11 Suzhou Institute of Systems Medicine, Suzhou, Jiangsu, China; 12 Center for Systems Medicine, Institute of Basic Medical Sciences, Chinese Academy of Medical Sciences, Peking Union Medical College, Beijing, China; 13 Lytix Biopharma AS, Gaustadalléen 21, Oslo, Norway; 14 Institute of Medical Biology, University of Tromsø, Tromsø, Norway; 15 Pôle de Biologie, Hôpital Européen Georges Pompidou, AP-HP, Paris, France; 16 Department of Women's and Children's Health, Karolinska Institute, Karolinska University Hospital, Stockholm, Sweden

## Abstract

LTX-315 is a cationic amphilytic peptide that preferentially permeabilizes mitochondrial membranes, thereby causing partially BAX/BAK1-regulated, caspase-independent necrosis. Based on the observation that intratumorally injected LTX-315 stimulates a strong T lymphocyte-mediated anticancer immune response, we investigated whether LTX-315 may elicit the hallmarks of immunogenic cell death (ICD), namely (i) exposure of calreticulin on the plasma membrane surface, (ii) release of ATP into the extracellular space, (iii) exodus of HMGB1 from the nucleus, and (iv) induction of a type-1 interferon response. Using a panel of biosensor cell lines and robotized fluorescence microscopy coupled to automatic image analysis, we observed that LTX-315 induces all known ICD characteristics. This conclusion was validated by several independent methods including immunofluorescence stainings (for calreticulin), bioluminescence assays (for ATP), immunoassays (for HMGB1), and RT-PCRs (for type-1 interferon induction). When injected into established cancers, LTX-315 caused a transiently hemorrhagic focal necrosis that was accompanied by massive release of HMGB1 (from close-to-all cancer cells), as well as caspase-3 activation in a fraction of the cells. LTX-315 was at least as efficient as the positive control, the anthracycline mitoxantrone (MTX), in inducing local inflammation with infiltration by myeloid cells and T lymphocytes. Collectively, these results support the idea that LTX-315 can induce ICD, hence explaining its capacity to mediate immune-dependent therapeutic effects.

Although cytotoxic chemotherapeutics used for the treatment of cancer often fail to achieve their ultimate goal – namely curing the patient in a permanent manner, without later relapse of the disease – there are a few examples in which conventional chemotherapy achieves long-term effects.^1, 2^ Beyond hematopoietic cancers, this applies for example to anthracycline-based adjuvant chemotherapy of breast cancer, which achieves a marked reduction in the relapse rate.^3^ The extraordinary success of this treatment might be explained by the fact that anthracyclines mobilize the immune system against malignant cells. Thus, cancer cells treated with anthracyclines *in vitro* elicits a T lymphocyte-mediated immune response against tumor-associated antigens when they are injected subcutaneously into immunocompetent mice, thereby protecting mice against rechallenge with live tumor cells of the same kind.^4, 5^ In other words, anthracyclines trigger immunogenic cell death (ICD).^6, 7, 8^

At the immunological level, it turned out that several pattern recognition receptors are involved in the recognition of dying cancer cells, meaning that their knockout or loss-of-function mutation abolishes the anticancer immune response. This applies for example to toll-like receptor 4 (TLR4) and formyl peptide receptor 1 (FPR1), meaning that anthracyclines have a reduced efficacy on tumors growing in *Tlr4*^−/−^ or *Fpr1*^−/−^ mice (as compared with WT mice) and that breast cancer patients bearing loss-of-function mutations in *TLR4* or *FPR1* have a comparatively poor prognosis after adjuvant chemotherapy with anthracyclines.^9, 10^ Neoadjuvant chemotherapy with anthracyclines causes a favorable change in the ratio between cytotoxic T lymphocytes and immunosuppressive regulatory T cells, in particular in those patients who manifest a complete pathological response.^11^ This constitutes a further proof in favor of the concept that anthracyclines mediate their antineoplastic effects via the induction of an anticancer immune response.

Anthracycline-induced ICD relies on one of the biochemical hallmarks of apoptosis, namely caspase activation. Thus, the pharmacological pan-caspase inhibitor Z-VAD-fmk, as well as transfection with the baculovirus inhibitor p35, do not interfere with anthracycline-induced cell death (which apparently can proceed in the absence of caspase activation), yet do abolish the immunogenicity of anthracycline-induced cell death.^4^ Mechanistic studies revealed that caspase inhibition interferes with several of the hallmarks of anthracycline-induced ICD, namely the exposure of calreticulin (CALR) on the cell surface,^5, 12^ as well as with the release of ATP that is usually associated with the blebbing phase of apoptosis.^13, 14^ CALR acts as a potent ‘eat-me' signal when it is exposed on the surface of stressed and dying cancer cells, facilitating the transfer of tumor antigens to dendritic cells.^15, 16, 17^ The mechanism of anthracycline-triggered CALR translocation to the cell surface is complex and involves the obligatory activation of caspase-8,^18, 19^ as well as the co-translocation of the disulfidisomerase PDIA3 (better known as ERp57).^20^ ATP acts as a potent chemoattractant, hence causing the influx of myeloid cells into the tumor bed.^21, 22^ ATP is released through a partially autophagy-dependent mechanism that also involves the caspase-3-mediated cleavage of pannexin-1 channels.^13, 21^ Removal of CALR (by knockdown) or extracellular ATP (by expression of the ATP-degrading ectoenzyme ENTPDI, better known as CD39) abolishes the immunogenicity of anthracycline-triggered cell death, similarly as does caspase inhibition.^5, 14^ On the basis of these results, we have been assuming that ICD was intimately linked to caspase activation and that necrosis (which does not involve caspase activation) would be intrinsically incompatible with ICD. Indeed, induction of necrosis by freeze-thawing of otherwise untreated cancer cells failed to yield an immunogenic preparation; and freeze-thawing actually destroyed the immunogenic properties of anthracycline-treated cells.^4^ Other hallmarks of ICD, namely the exodus of HMGB1 from the nuclei of dead cells and the induction of a type-1 interferon response occur in a largely caspase-independent manner.^9, 23^

In apparent contradiction with our findings, Patricia Agostinis found that photodynamic therapy stimulates ICD without capase-8 activation,^24^ establishing the existence of another type of ICD (called ‘type-2 ICD' as opposed to the anthracycline-induced ‘type-1 ICD') that differs in its signaling mechanisms.^6, 25^ In accord with this possibility, artificial activation of necroptosis by chemical dimerization of a modified transgenic RIPK3 (better known as RIP3) protein can induce ICD.^26^ Hence, cell death that does not involve caspase activation can be perceived as immunogenic. Nonetheless, this cell death triggered by photodynamic therapy or RIP3 activation does exhibit all major signs of ICD including CALR exposure, ATP release, and HMGB1 exodus.^24, 26^

There is yet additional, though indirect evidence that necrosis may be immunogenic. Thus, LTX-315, a cationic amphiphilic peptide derivative that permeabilizes inner mitochondrial membranes and induces necrosis,^27, 28^ is a strong inducer of anticancer immune responses if injected into tumors developing on immunocompetent mice.^29, 30^ Indeed, intratumoral injection of LTX-315 can lead to the eradication of B16 melanomas, accompanied by an immune response that protects cured mice against rechallenge with live B16 cells.^29, 30^ Challenged by this observation, we investigated whether LTX-315 can trigger ICD by characterizing the hallmarks of ICD (CALR exposure, ATP release, HMGB1 exodus and type-1 interferon response). Our results indicate that LTX-315 can induce all characteristics of ICD in a caspase-independent manner *in vitro*. When injected *in vivo*, into tumors, LTX-315 induces massive focal necrosis, followed by immune infiltration.

## Results and Discussion

### LTX-315 induces calreticulin exposure

**Figure 1:**
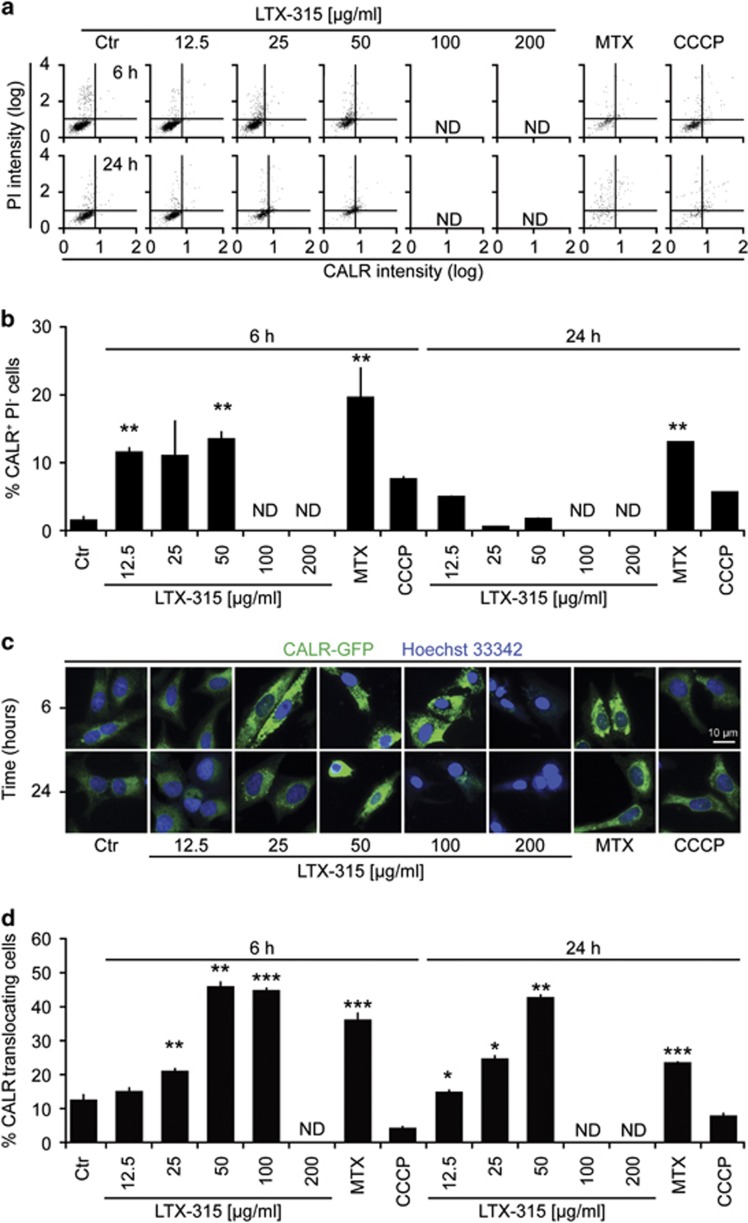
Induction of CALR exposure by LTX-315. (**a, b**) U2OS cells were treated for the indicated period (6 or 24 h) with LTX-315, MTX, or CCCP. Cells were either subjected to surface immunofluorescence staining to detect CALR in viable, PI-excluding cells (representative cytofluorometry pictograms in a and statistical analyses in **b**). Note that high concentration of LTX-315 killed >99% of the cells, meaning that the fraction of PI-negative cells was too low to appreciate CRT exposure among them. (**c, d**) U2OS cells stably expressing a calreticulin-GFP fusion protein were treated with the indicated agents, fixed at the indicated time points, counterstained with the chromatin dye Hoechst 33342 and subjected to automatic fluorescence microscopy (representative microphotographs in **c** and statistical analyses in **d**). Quantitative results are shown as means±S.D. of triplicates. Asterisks indicate significant differences (unpaired Student's *t*-test) with respect to untreated controls. *P<0.05; **P<0.01; ***P<0.001

When added to U2OS osteosarcoma cells, LTX-315 caused the induction of CALR exposure, as detectable by immunofluorescence staining of viable (propidium iodide-negative, PI^−^) cells. This effect was detectable at LTX-315 doses that have no major cytotoxic effect (12.5  and 25 *μ*g/ml) as well around the half-lethal dose (50 *μ*g/ml) and was particular strong at 6 h of treatment (Supplementary Figure 1). However, at a later time point (24 h) no viable cells exposing CALR were detectable (Figures 1a and b). Previous studies indicate that the aggregation of CALR precedes its translocation to the plasma membrane.^31^ U2OS cells stably transfected with a CALR-green fluorescent protein (GFP) fusion protein manifested the dose-dependent aggregation of CALR-GFP in cytoplasmic granules after treatment with LTX-315 that was detectable at 6 h, as well as at 24 h (when the cells containing aggregated CALR-GFP probably have lost their viability) (Figures 1c and d). Interestingly, LTX-315 failed to induce the phosphorylation of eukaryotic initiation factor 2*α* (eIF2*α*) in conditions in which the positive control (induction of endoplasmic reticulum stress with the SERCA pump inhibitor thapsigargin (THAP)) yielded a major response (Supplementary Figure 2). These results indicate that LTX-315 can stimulate CALR exposure through a pathway that does not involve eIF2*α* phosphorylation, hence resembling that induced by photodynamic therapy.^24^

### LTX-315 induces the release of ATP and HMGB1

**Figure 2:**
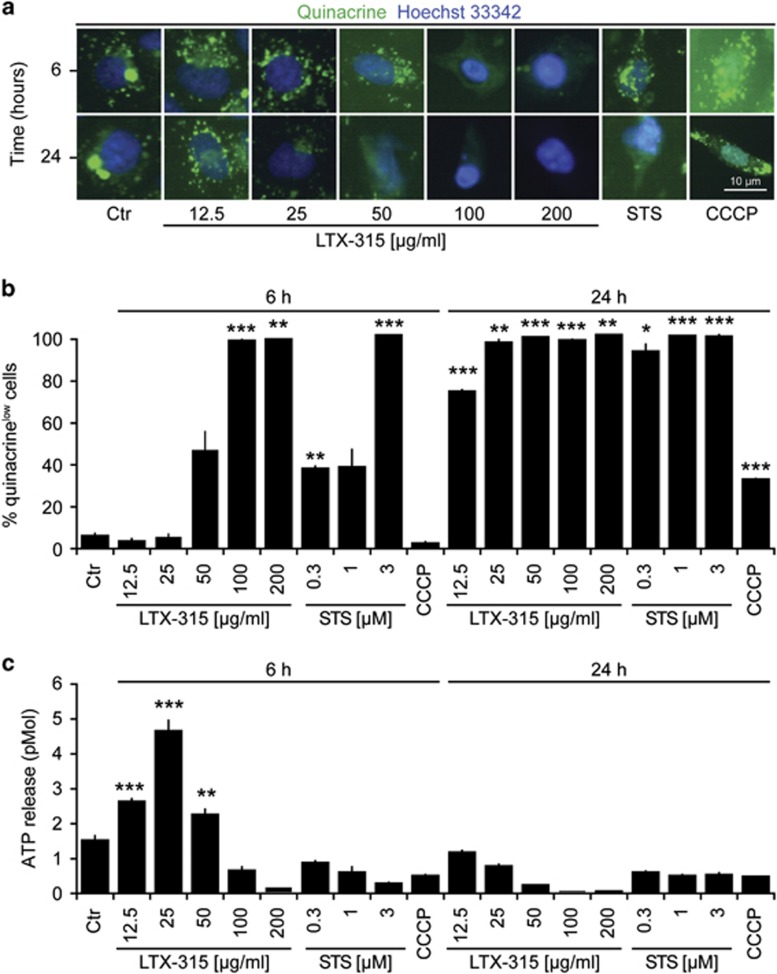
Release of ATP from cells exposed to LTX-315. U2OS cells were cultured in the indicated conditions with LTX-315, staurosporin (STS), or CCCP. Then, live cells were stained with quinacrine and immediately analyzed by fluorescence microscopy. Representative images are shown in (**a**). Quantitative results (means±S.D. of triplicates) are provided in (**b**). Furthermore, extracellular ATP was measured in the culture supernatant, as shown in (**c**). Asterisks indicate significant differences (unpaired Student's *t*-test) with respect to untreated controls. *P<0.05; **P<0.01; ***P<0.001

**Figure 3:**
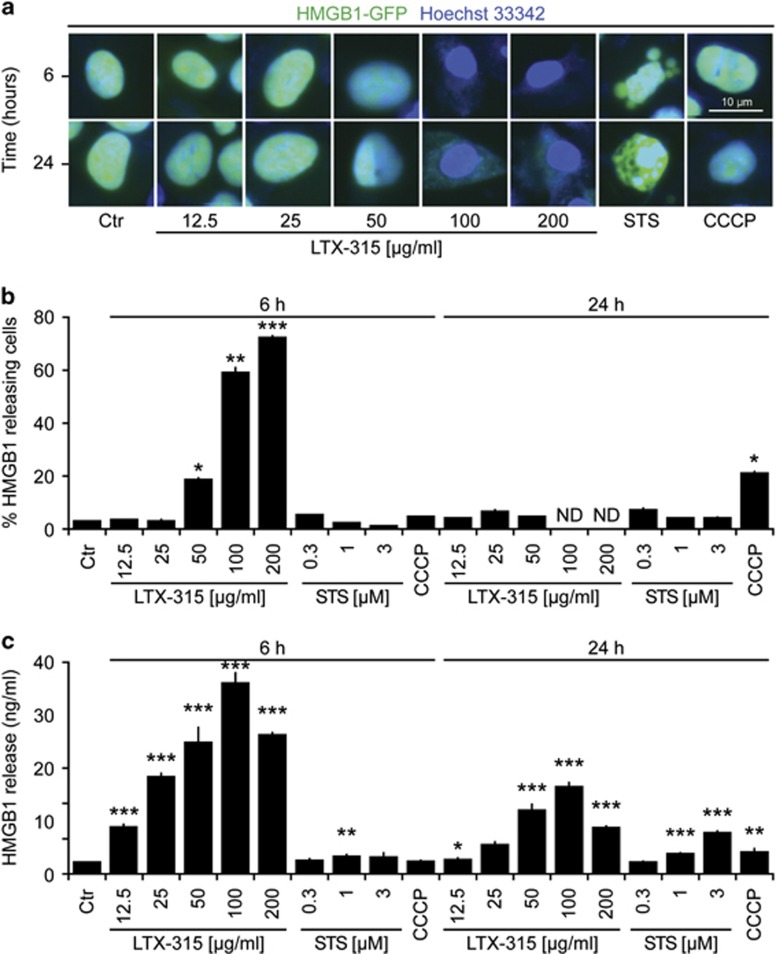
Release of nuclear HMGB1 from cells exposed to LTX-315. U2OS cells stably expressing an HMGB-GFP fusion protein were cultured in the indicated conditions with LTX-315, staurosporin (STS), or CCCP. Cells then were fixed and subjected to automatic fluorescence microscopy. Representative images are provided in (**a**). Quantitative results (means±S.D. of triplicates) are given in (**b**). Alternatively, parental U2OS cells were subjected to a similar treatment, followed by detection of HMGB1 in the culture supernatant, as shown in (**c**). Asterisks indicate significant differences (unpaired Student's *t*-test) with respect to untreated controls. *P<0.05; **P<0.01; ***P<0.001

We next investigated whether LTX-315 would be able to stimulate the release of the two DAMPs ATP and HMGB1 from cancer cells. For this, we first determined the capacity of LTX-315 to reduce the staining of U2OS cells with quinacrine, a fluorophore that selectively stains ATP-rich cytoplasmic vesicles. LTX-315 caused the depletion of quinacrine-positive vesicles (Figures 2a and b). Moreover, it provoked an increase in extracellular ATP levels, as measured by means of a bioluminescence reaction (Figure 2c). U2OS cells stably expressing an HMGB1-GFP fusion protein in their nuclei exhibited loss of the fluorescent signal upon short-term culture (6 h) with LTX-315. At later time points, the few adherent cells found in the cultures retained the HMGB1-GFP-dependent fluorescent signal (Figures 3a and b). The release of HMGB1 into the culture supernatant by cells treated with LTX-315 could be confirmed by means of a sensitive ELISA (Figure 3c). In conclusion, LTX-315 can stimulate the release of both DAMPs from cancer cells.

### LTX-315 stimulates a type-1 interferon response

**Figure 4:**
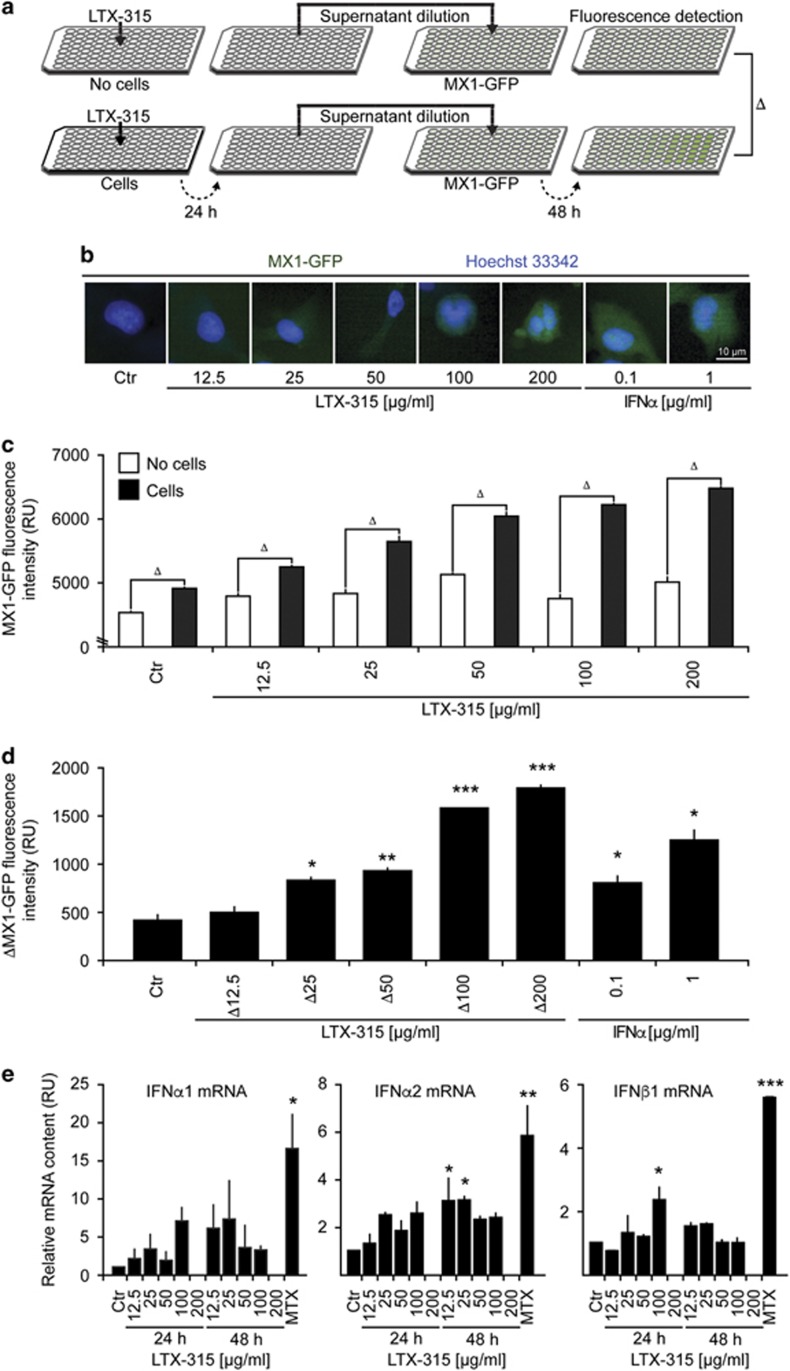
Induction of a type-1 interferon response by LTX-315. (**a–d**) A schematic representation of the experimental design is shown in (**a**). LTX-315 was added at variable concentrations to culture media (without cells, above) or U2OS cell cultures (below). Recombinant IFN-*α*1 was used as a positive control. After 24 h, the culture supernatants were recovered and added to fresh cultures (1:16 dilution) of U2OS cells stably expressing GFP under the MX1 promoter (MX1-GFP). After an additional 48-h culture period, cells were fixed, counterstained with Hoechst 33342, and subjected to automated fluorescence microscopy and image analysis. Representative images are shown in (**b**), raw data of quantitations (means±S.D. of triplicates) in (**b**), and the subtraction of initially cell-free versus-cell-containing cultures in (**d, e**). Detection of type-1 interferons by RT-PCR. Cells were incubated as indicated with variable amounts of LTX-315 for distinct periods and then subjected to mRNA extraction and RT-PCR. Asterisks indicate significant differences (unpaired Student's *t-*test) with respect to untreated controls. *P<0.05; **P<0.01; ***P<0.001

ICD inducers can stimulate the production of type-1 interferon, which act on type-1 interferon receptors (IFNAR) to stimulate the transcription of a series of target genes including that of *MX1* (which codes for myxovirus (influenza) resistance 1, MX1).^32^ The primary event leading to this type-1 interferon response resides in the stimulation of toll-like receptor-3 (TLR3) by extracellular nucleic acids.^33^ To reveal this phenomenon, we created a cell line expressing GFP under the control of the *MX1* promoter. Such cells are highly reactive to the prototypical type-1 interferon, interferon-*α*1 (IFN*α*1), and hence become GFP positive. The conditioned medium of LTX-315-treated cells also was able to stimulate GFP expression by such biosensor cell lines, more so than LTX-315-containing culture media (Figures 4a–d). In addition, we found that LTX treatment of cells induced the expression of mRNAs encoding several type-1 interferons including IFN*α*2 and IFN*β*1 (Figure 4e). We conclude from these results that LTX-315 can stimulate a type-1 interferon response.

### LTX-315 induces tumor necrosis and immune infiltration

**Figure 5:**
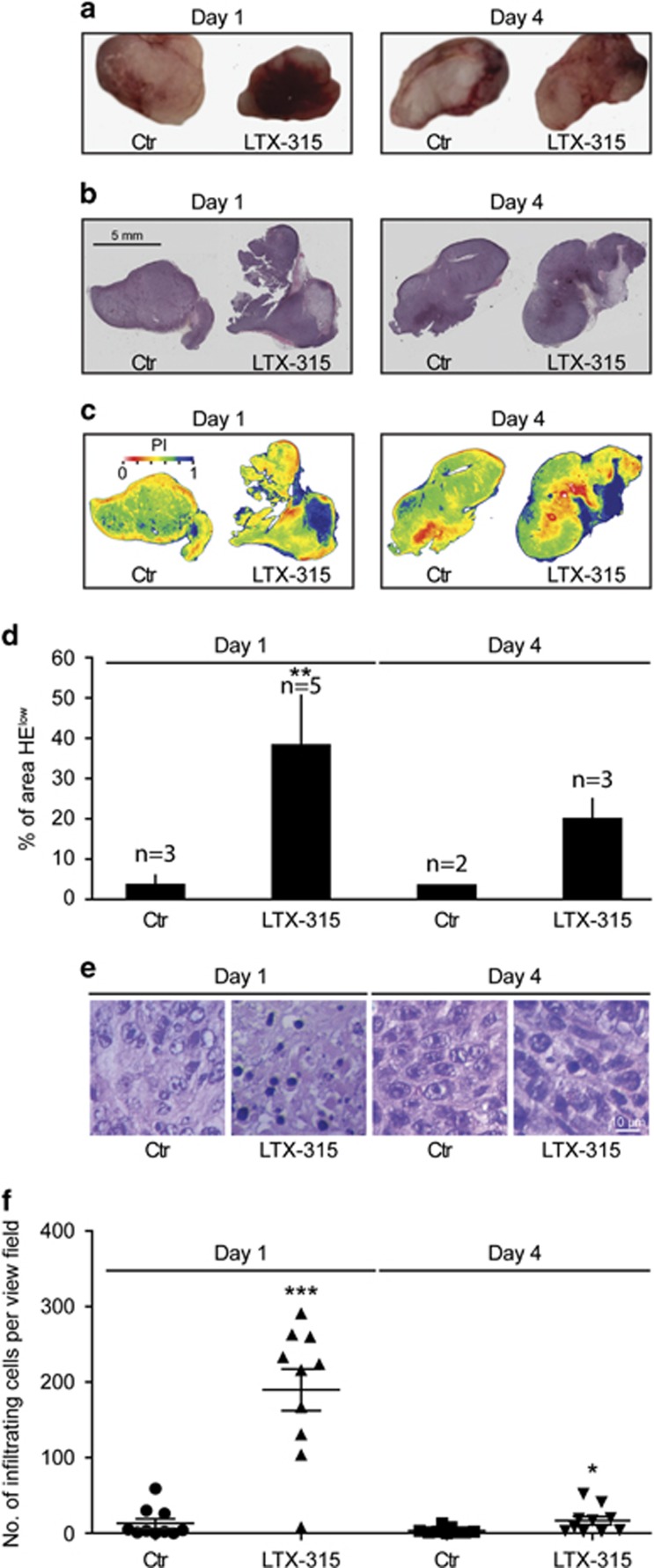
Macroscopic and microscopic signs of necrosis induced by LTX-315 *in vivo*. MCA205 fibrosarcoma was established on C57/Bl6 mice and injected with PBS (control, Ctr) or LTX-315. Tumors were harvested 1 or 4 days later and were either photographed after excision to document their macroscopic appearance (**a**) or subjected to HE staining (raw appearance in (**b**) and ratio of eosin over hematoxylin in (**c**)) to quantify the area with low hematoxylin staining (**d**). Results in (**d**) are means ±S.E.M. for the indicated number of tumors. Representative HE staining patterns of necrotic areas are shown in (**e**) and the number of infiltrating leukocytes per view field were determined in (**f**). Asterisks indicate significant differences (unpaired Student's *t-*test) with respect to PBS-treated controls. *P<0.05; **P<0.01; ***P<0.001

**Figure 6:**
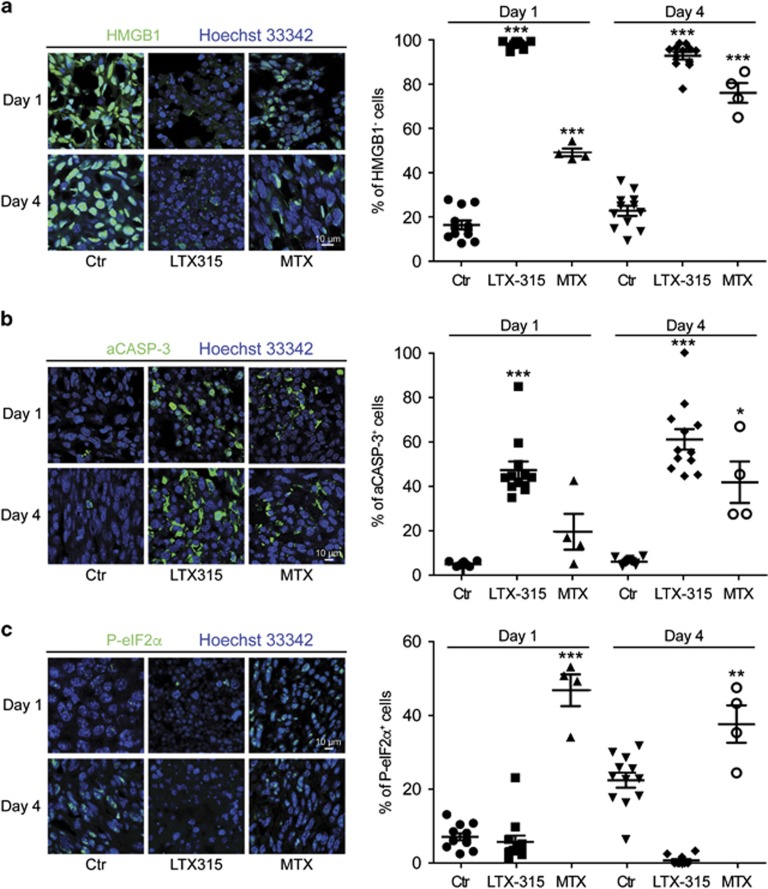
Cell death and stress induced by LTX-315 *in vivo*. Subcutaneous MCA205 fibrosarcomas were injected locally with PBS (control, Ctr), LTX-315, or MTX. One or four days later, tumor was retrieved and subjected to immunofluorescence staining to detect HMGB1 (**a**), activated caspase-3 (**b**), or phosphorylated eIF2*α* (**c**). Representative staining patterns are shown on the left and quantitative results (each dot represents one view field) are shown on the right. Asterisks indicate significant differences (unpaired Student's *t-*test) with respect to PBS-treated controls. *P<0.05; **P<0.01; ***P<0.001

**Figure 7:**
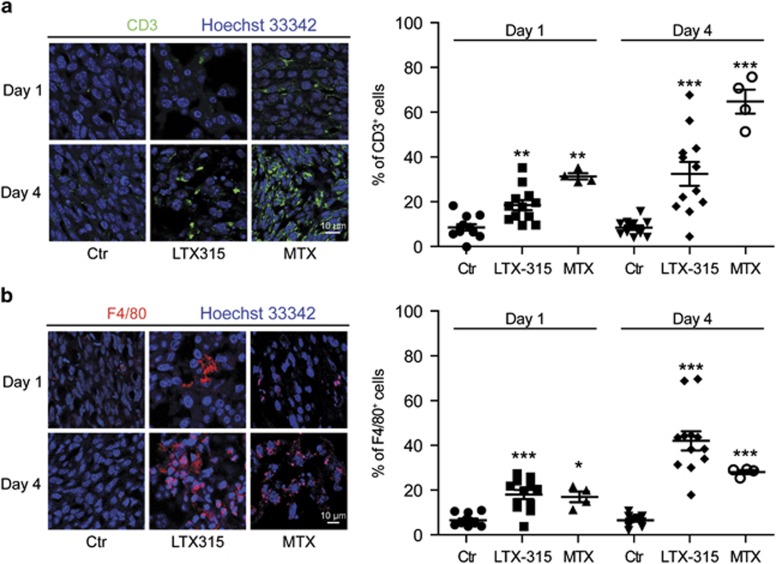
Immune infiltrates induced by LTX-315 *in vivo*. Subcutaneous MCA205 fibrosarcomas were injected locally with PBS (control, Ctr), LTX-315, or MTX. One or four days later, tumor was excised and subjected to immunofluorescence detection of CD3 (**a**) or F4/80 (**b**). Representative staining patterns are represented on the left and quantitative results (each dot represents one view field) are shown on the right. Asterisks indicate significant differences (unpaired Student's *t*-test) with respect to PBS-treated controls. *P<0.05; **P<0.01; ***P<0.001

To further investigate the capacity of LTX-315 to stimulate an anticancer immune response, we injected the product into established MCA205 fibrosarcomas growing on C57Bl/6 mice. The injection of LTX-315 caused hemorrhagic necrosis that, by macroscopic detection, was particularly detectable 1 day post injection (as opposed to 4 days post injection) (Figure 5a). LTX-315 also caused persistent necrosis, which was detectable as a de-coloration of the hematoxylin eosin (HE) staining (Figures 5b–d). Within this necrotic area, we found a major influx of immune/inflammatory cells with a lymphocyte-like morphology that was particularly strong 1 day after LTX-315 injection (Figures 5e and f). Immunofluorescence staining revealed that the necrotic areas had lost all HMGB1 (Figure 6a; Supplementary Figure 3). Surprisingly, a substantial fraction of the cells in the necrotic area exhibited positivity for activated caspase-3 (CASP-3a), in spite of the fact that their nuclei rarely had a shrunken or fragmented morphology compatible with apoptosis (Figure 6b). In contrast to anthracyclines, which induce a major phosphorylation of eIF2*α*, LTX-315 failed to cause any immunofluorescence-detectable sign of eIF2*α* phosphorylation (Figure 6c). However, LTX-315 and MTX were similar in their capacity to provoke the influx of CD3^+^ T cells and F4/80^+^ macrophages into the tumor (Figures 7a and b). Hence, LTX-315 triggers a local inflammatory and immune response that is comparable in its magnitude to that induced by anthracyclines.

### Concluding remarks

In this study, we present ample evidence that LTX-315 can induce all hallmarks of ICD including CALR exposure, ATP release, HMGB1 exodus and type-1 IFN responses when added to cultured cancer cells. The lethal mode of action of LTX-315 has recently been elucidated.^27, 28^ LTX-315 preferentially enriches at mitochondrial membranes, causing their permeabilization, meaning that the barrier function of both the inner and the outer mitochondrial membrane is lost. Cells lacking the two pro-apoptotic multidomain proteins from the BCL2 family, BAX and BAK, were slightly less susceptible to LTX-315-mediated killing than were control cells, although this protection was only partial. Moreover, cells engineered to lose their mitochondria (by transfection with Parkin followed by treatment with a protonophore causing complete removal of the organelles by mitophagy) became relatively resistant against LTX-315, although this effect was rather partial as well.^27, 28^ In contrast, inhibition of caspases failed to subvert the cytotoxic effects of LTX-315. Moreover, MCA205 cells used in the *in vivo* experiments shown in this paper are fully susceptible to killing by LTX-315, in spite of the fact that they lack expression of the essential necroptotic signaling molecule RIP3. Similarly, knockout of the necroptotic effector molecule RIP3 did not affect the dose response of cell death induction in TC1 lung cancer cells (Supplementary Figure 4). Altogether, these data are compatible with the hypothesis that LTX-315 essentially causes passive cell death, without any major active contribution of lethal signal transduction pathways that are usually participating in the dismantling phases of apoptosis or necroptosis.^34, 35^

We previously demonstrated that killing cancer cells by repeated freeze-thawing, which induces necrotic destruction, fails to yield an immunogenic preparation,^4^ and this finding has been confirmed in other experimental systems.^26^ In sharp contrast, induction of necroptosis by chemical dimerization of a modified RIP3 molecule can induce ICD, a phenomenon that involves the RIP1-dependent activation of an NF-*κ*B-led transcriptional program.^26^ Here, we show that LTX-315 also can induce ICD in the context of a necrotic pathway that is independent of RIP3 (and is not inhibited by necrostatin 1, which inhibits RIP1),^27, 28^ suggesting that different pathways leading to necrosis can cause ICD. Obviously, the release of ATP from cells cannot be dictated any more by caspases and rather must occur in a passive manner (instead of the caspase-dependent activation of Pannexin 1 channels).^13, 14^ Nonetheless, we have not yet explored the question as to whether the transcription of NF-*κ*B-dependent genes would contribute to the immunogenicity of LTX-315-induced cell death. Several physical methods, including photodynamic therapy, high hydrostatic pressure, and hyperthermia, have been developed for the necrotic destruction of cancer cells and actually can induce ICD.^36, 37^ Altogether, these results suggest that freeze-thawing may actually constitute an exception among necrosis inducers with regard to its incapacity to induce ICD. This might be related to the full destruction of cells and subcellular structures by microcrystals generated during this process (‘cryodamage') with the subsequent activation of hydrolases that may destroy DAMPs or antigens required for immunogenicity.

In cell cultures, LTX-315 induces ‘pure' necrosis, meaning that it fails to stimulate the appearance of the morphological and biochemical hallmarks of apoptosis (nuclear shrinkage or fragmentation, activation of effector caspases).^27, 28^ When injected into tumors, it causes a focal hemorrhagic necrosis that is rapidly filled by inflammatory cells including macrophages and T cells. Hence, local injection of LTX-315 into the tumor is as efficient as MTX in stimulating a local inflammatory/immune reaction. At difference with the *in vitro* results, however, LTX-315-injected tumor sites accumulate cells that stain with an antibody recognizing active, proteolytically mature caspase-3. At present, the apparent discrepancy between the incapacity of LTX-315 to induce caspase-3 activation *in vitro* and its *in vivo* effects is difficult to understand. The frequency of cells staining for active caspase-3 is high, suggesting that the phenomenon must occur in cancer (as opposed to stromal and inflammatory) cells. Possibly, the *in vivo* effects of LTX-315 are affected by altered pharmacokinetics, as suggested by the fact that LTX-315-induced killing is inhibited by high serum concentrations (Supplementary Figure 5). However, high serum did not switch LTX-315-induced necrosis to apoptosis, suggesting that other hitherto unknown factors must contribute to LTX-315-induced caspase-3 activation. As a possibility, DAMPs released from a fraction of cells succumbing to LTX-315 through necrosis might trigger apoptosis in adjacent cells.^38^ This possibility is under active investigation in our laboratories.

Altogether, our present work demonstrates the possibility that stimuli that *a priori* induce necrosis can stimulate local immune reactions that ignite a strong anticancer immune response. It will be interesting to explore the possibility of rendering LTX-315-like molecules more specific, namely by fusing them to targeting peptides, hence facilitating their uptake by malignant (rather than normal) cells. Moreover, it will be important to compare peptides preferentially lysing distinct organelles for their ICD-triggering capacity.

## Materials and Methods

### Cell culture and treatment

Unless otherwise indicated, chemicals were obtained from Sigma-Aldrich (St. Louis, MO, USA). LTX-315 was obtained from Lytix Biopharma (Oslo, Norway). Culture media were from Gibco-Invitrogen (Carlsbad, CA, USA). U2OS cells were obtained from the American Type Culture Collection (ATCC) and cultured under standard cell culture conditions (5% CO_2_; 37 °C) in a water-saturated atmosphere within a cell culture incubator (HeraCell, Heraeus, Germany). U2OS that were genetically engineered as described,^31^ were cultured in DMEM GlutaMax supplemented with 10% FBS. In short, cells were stably transduced or transfected with cDNA vectors coding for the indicated fluorescent fusion proteins. Subsequently, stable expressing cells were selected by means of appropriate selection antibiotics and clones were obtained by single cell sorting using a FACS DIVA (Becton Dickinson, Franklin Lakes, NJ, USA). Unless otherwise indicated, staurosporin was used at 1 *μ*M, carbonyl cyanide m-chlorophenyl hydrazine (CCCP) at 100 *μ*M, MTX at 1 *μ*M, and THAP at 1 *μ*M.

### Interferon biosensor

For the detection of interferon-mediated responses, U2OS cells were transfected with pEZX-PF02 (Genecopoeia, Rockville, MD, USA) coding for eGFP under the control of the myxovirus (influenza virus) resistance 1 (MX1) promoter. Stably expressing cells were selected using puromycin and clones were generated by single cell sorting using a FACS DIVA (Becton Dickinson).

### Automated image acquisition

One day before the experiment, 4 × 10^3^ cells were seeded in tissue culture-treated 96-well μClear imaging plates (Greiner BioOne, Frickenhausen, Germany) and incubated under standard tissue culture conditions. On the following day, the cells were treated, and after incubation for 6 or 24 h the cells were fixed with 3.7% formaldehyde solution containing 1 *μ*M Hoechst 33342 overnight at 4 °C. The fixative was changed to PBS, and the plates were subjected to automated image analysis. For automated fluorescence microscopy, a robot-assisted Molecular Devices IXM XL BioImager (Molecular Devices, Sunnyvale, CA, USA) equipped with Sola light sources (Lumencor, Beaverton, OR, USA), adequate excitation and emission filters (Semrock, Rochester, NY, USA), and a 16-bit monochromes sCMOS PCO.edge 5.5 camera (PCO, Kelheim, Germany) and a × 20 PlanAPO objective (Nikon, Tokyo, Japan) was used to acquire six view fields/well, followed by image processing with the custom module editor of the MetaXpress software (Molecular Devices). Depending on the utilized biosensor cell line, the primary region of interest (ROI) was defined by a polygon mask around the nucleus allowing for the enumeration of cells and (in the case of HMGB1-GFP expressing cells) the quantification of nuclear HMGB1. Cellular debris was excluded from the analysis, and secondary cytoplasmic ROIs were used for the quantification of CALR-GFP or quinacrine containing vesicles. For the latter, the images were segmented and analyzed for GFP granularity by comparing the standard deviation of the mean fluorescence intensity of groups of adjacent pixels within the cytoplasm of each cell to the mean fluorescence intensity in the same ROI using the MetaXpress software (Molecular Devices).

### Tissue immunohistochemistry and confocal imaging

Tumors upon dissection were fixed with 3.7% of PFA for 4 h at ambient temperature. Then, the tissue was incubated in 30% sucrose solution for 24 h at 4 °C. One half of the tumor was used for hematoxylin eosin staining according to standard procedures, and the other half was used for immunohistochemistry (IHC). The tissue was cut in 6-*μ*m-thick slices using a Cryostat CM (Leica, Mannheim, Germany) and was then incubated with blocking buffer (10% FBS in PBS) for 30 min at RT and stained with primary antibodies and secondary antibody-fluorochrome conjugates both diluted in blocking buffer according to standard procedures. Coverslips were mounted in DAPI containing Fluomount-G (Southernbiotech, Birmingham, AL, USA). Images were acquired using a TCS SP 8 confocal microscope (Leica) equipped with a × 40 oil objective (Nikon). The percentage of phenotypically altered cells was evaluated using imageJ (http://imagej.nih.gov/ij/).

### Determination of surface exposed CALR by immunofluorescence

Cells were collected and washed twice with cold PBS. Following the cells were incubated with an anti-CALR antibody (ab2907; Abcam, Cambridge, UK) diluted in cold blocking buffer (1% BSA in PBS) for 60 min on ice, followed by washing and incubation with AlexaFluor 488-conjugates (Invitrogen) in blocking buffer (for 30 min). Thereafter, cells were washed in cold PBS, PI was added to the final concentration of 1 *μ*g/ml, and samples were analyzed by means CyAn ADP (Beckman Coulter) coupled to a Hypercite autosampler (IntelliCyte; Albuquerque, NM, USA). The analysis was limited to living (PI^−^) cells. Data were statistically evaluated using the R software (https://www.r-project.org).

### Assessment of ATP secretion

For the detection of ATP containing vesicles, the cells were labeled with quinacrine as described.^12^ In short, cells were incubated with 5 *μ*m quinacrine and 1 *μ*g/ml Hoechst 33342 in Krebs-Ringer solution (125 mM NaCl, 5 mM KCl, 1 mM MgSO_4_, 0.7 mM KH_2_PO_4_, 2 mM CaCl_2_, 6 mM glucose and 25 mM Hepes, pH 7.4) for 30 min at 37 °C. Thereafter, cells were rinsed with Krebs-Ringer and living cells were microscopically examined. Alternatively, the concentration of extracellular ATP was assessed by means of the ENLITEN ATP assay (Promega, Madison, WI, USA), based on luciferin conversion, following the manufacturer's instructions. Chemoluminescence was measured using an i3 Paradigm multi-label reader (Molecular Devices).

### Determination of extracellular HMGB1 concentration

Quantification of HMGB1 in cell supernatants was performed by means of an enzyme-linked immunosorbent assay (HMGB1 ELISA kit II, Shino Test Corporation, Tokyo, Japan) following the manufacturer's instructions. Absorbance was analyzed by means of an i3 Paradigm multi-label reader (Molecular Devices).

### Quantitative real-time PCR for interferon production

Total RNA extraction and genomic DNA removal were performed with the GeneJet RNA purification kit (ThermoScientific, Waltham, MA, USA) following the manufacturer's instructions. Total RNA (5 *μ*g from each sample) was reverse-transcribed into cDNA with the Maxima first-strand cDNA synthesis kit (ThermoScientific) in the presence of random primers and Deoxynucleoside Triphosphate (Invitrogen, Carlsbad, CA, USA). Expression of IFN-related genes was analyzed by means of the Power SYBR Green PCR Master Mix (ThermoScientific) mixed with primers (Invitrogen) on a StepOnePlus Real-Time PCR System (ThermoScientific). qRT-PCR data were invariably normalized to the expression levels of the housekeeping gene HPRT1.

### Animals

Six- to seven-week-old female wild-type C57Bl/6 mice obtained from Harlan France (Gannat, France) were maintained in the animal facility of the Gustave Roussy Campus Cancer in specific pathogen-free conditions in a temperature-controlled environment with 12 h light, 12 h dark cycles and received food and water *ad libitum*. Animal experiments were in compliance with the EU Directive 63/2010 and protocols. Protocols 2013_094A were approved by the Ethical Committee of the Gustave Roussy Campus Cancer (CEEA IRCIV/IGR no. 26, registered at the French Ministry of Research). MCA205 tumors were estabished in C57Bl/6 host by injecting 500 000 cells subcutaneously. When tumor became palpable, 300 *μ*g of LTX-315 was injected intratumorily, and tumors were recovered 1 or 4 days post injection.

### Statistical analyses

Unless otherwise specified, experiments were performed in quadruplicates. Data were analyzed with the freely available software R (https://www.r-project.org). Statistical significance was calculated using a two-tailed Student's *t*-test with Welch correction, and *P-*values <0.05 were considered as statistically significant. Thresholds for each assay were applied based on the Gaussian distribution of positive and negative controls.

## Abbreviations

ATCC: American Type Culture Collection
ATP: adenosine triphosphate
BAK1: BCL2-antagonist/killer 1
BAX: BCL2-associated X protein
CALR: calreticulin
CASP: caspase
DAMP: damage-associated molecular pattern
DAPI: (4',6-diamidino-2-phenylindole)
eIF2*α*: eukaryotic translation initiation factor 2*α*
ENTPD1: ectonucleoside triphosphate diphosphohydrolase 1
FBS: fetal bovine serum
FPR1: formyl peptide receptor 1
GFP: green fluorescent protein
HMGB1: high mobility group box 1
ICD: immunogenic cell death
IFN: interferon
MX1: myxovirus (influenza virus) resistance 1
PDIA3: protein disulfide isomerase family A member 3
PFA: paraformaldehyde
PI: propidium iodide
RIPK: receptor interacting serine/threonine kinase
ROI: region of interest
RT-PCR: real-time PCR
TLR: Toll-like receptor
