# Supplementary figures and images for: The oncolytic peptide LTX-315 triggers immunogenic cell death
Source: Cell Death Dis. 2016 Mar 10;7(3):e2134–. doi: 10.1038/cddis.2016.47 (PMC4823948; doi:10.1038/cddis.2016.47)

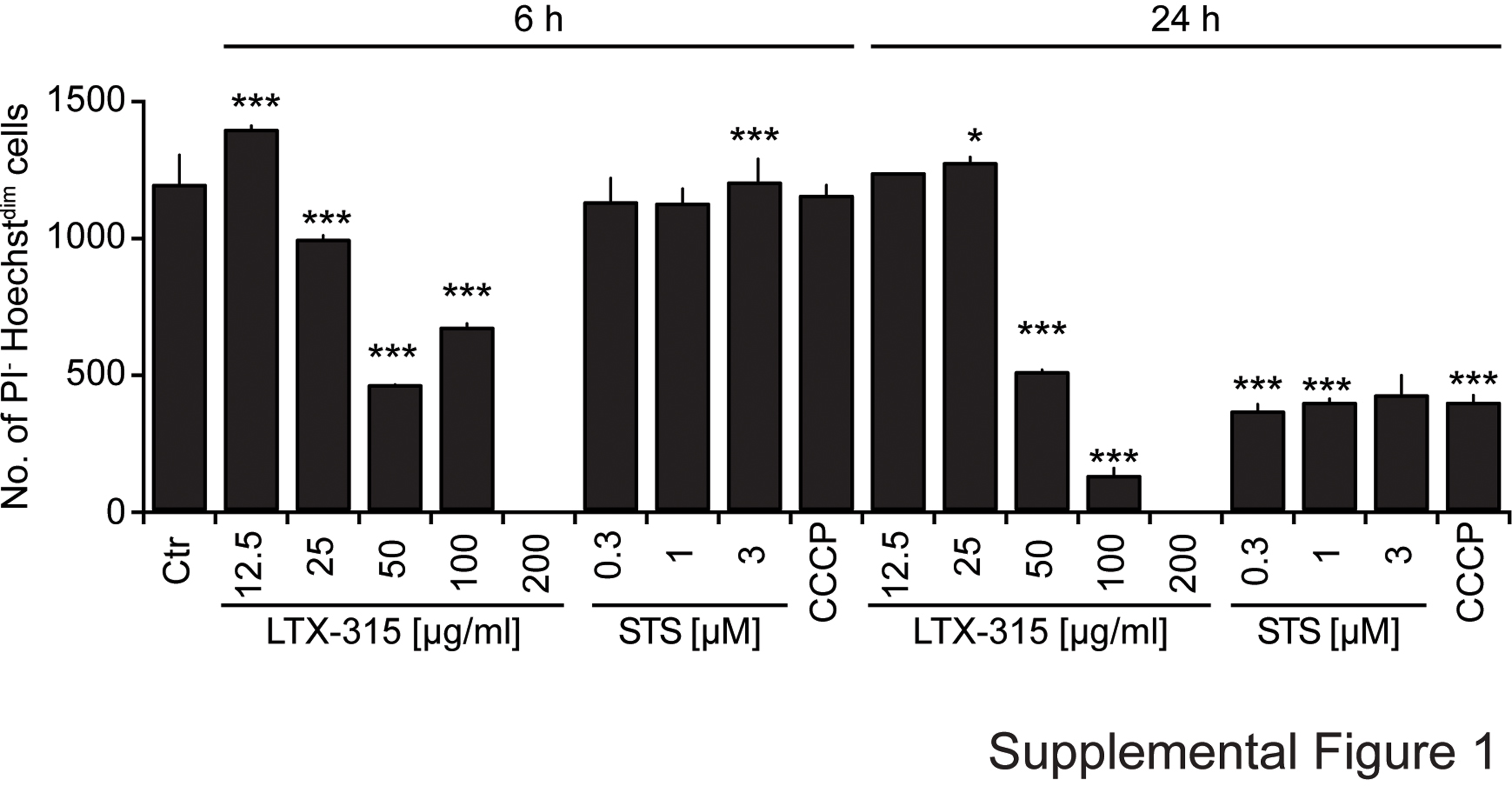

Supplement: Supplementary Figure 1 [file cddis201647x1.tif]

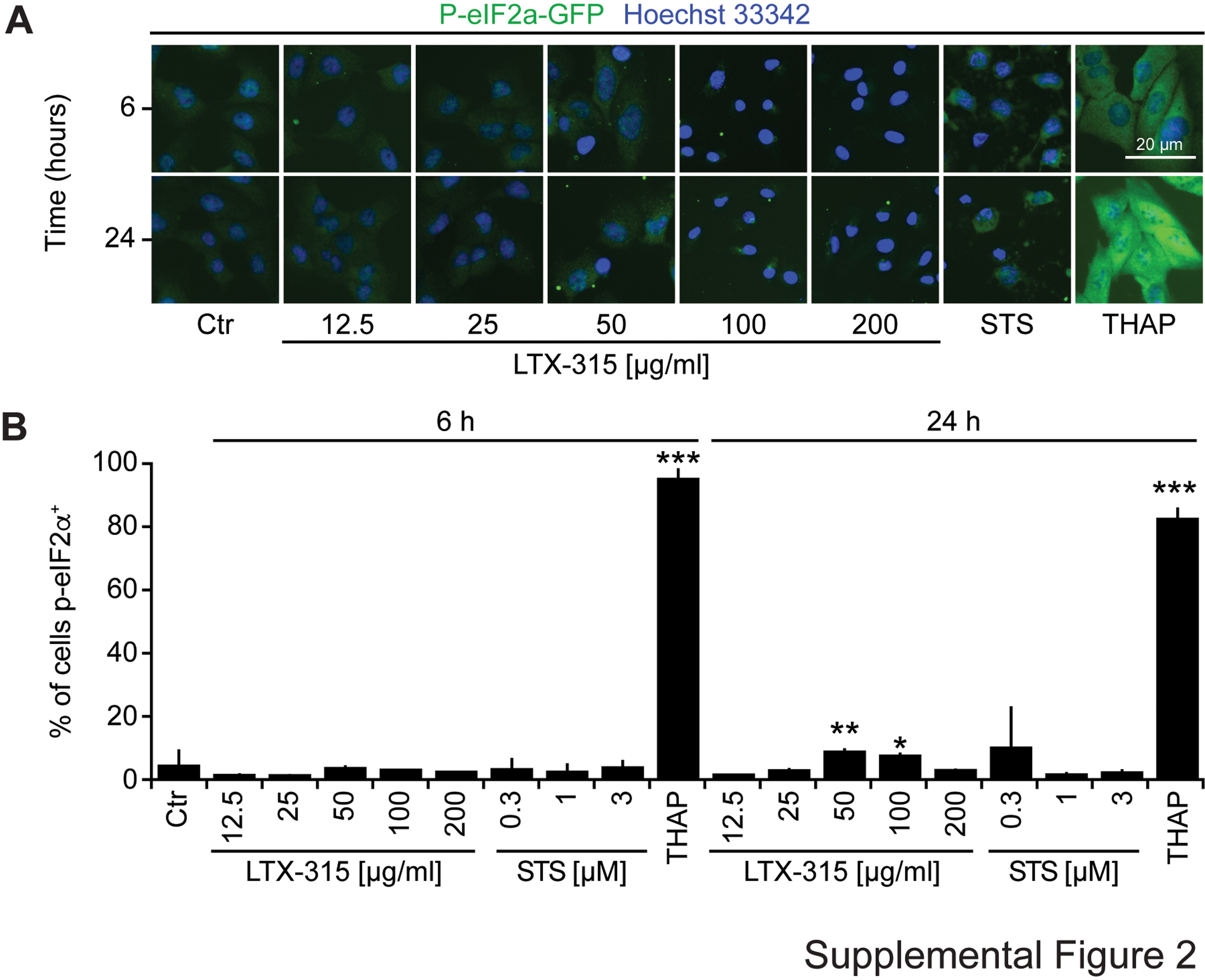

Supplement: Supplementary Figure 2 [file cddis201647x2.tif]

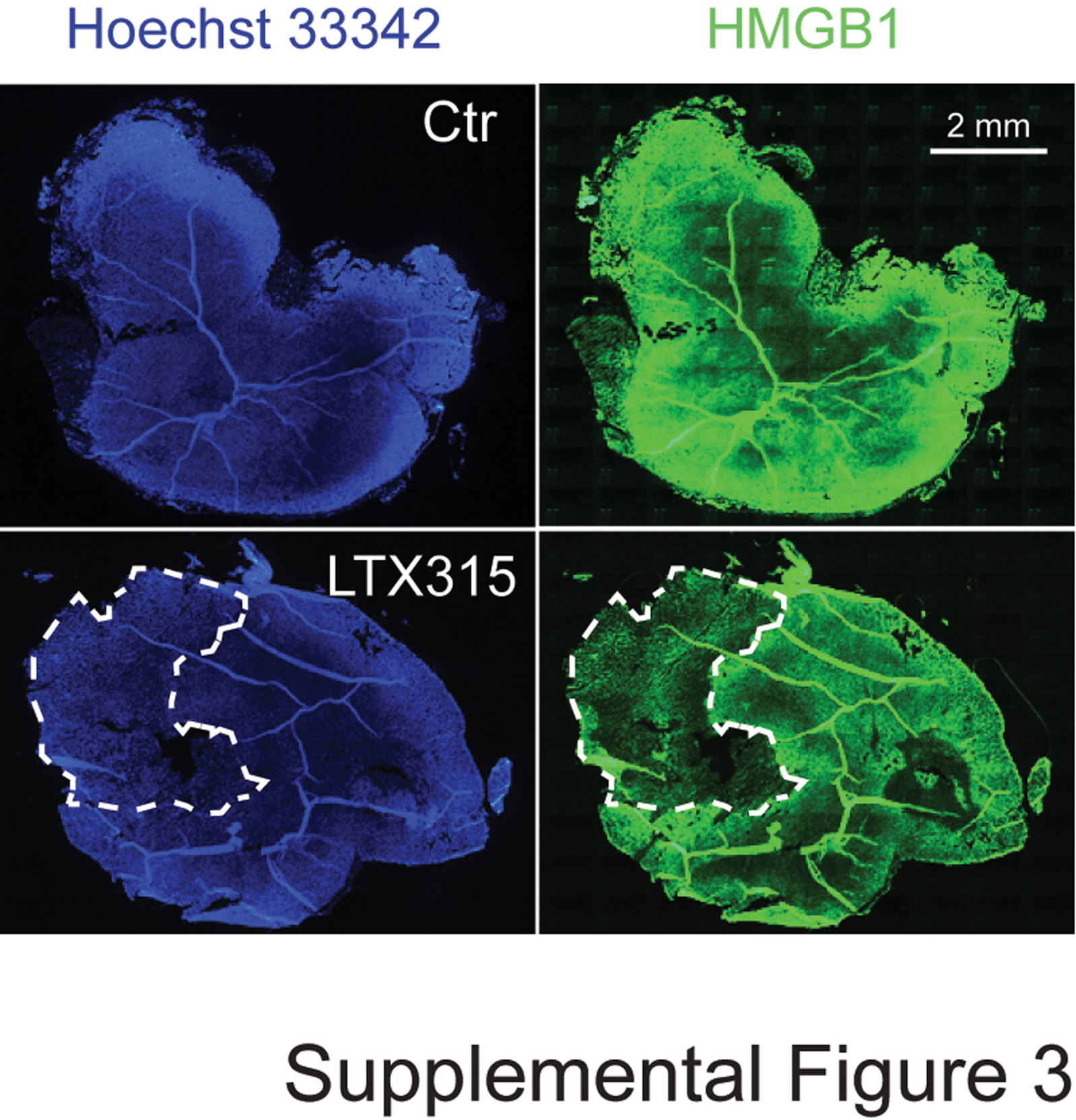

Supplement: Supplementary Figure 3 [file cddis201647x3.tif]

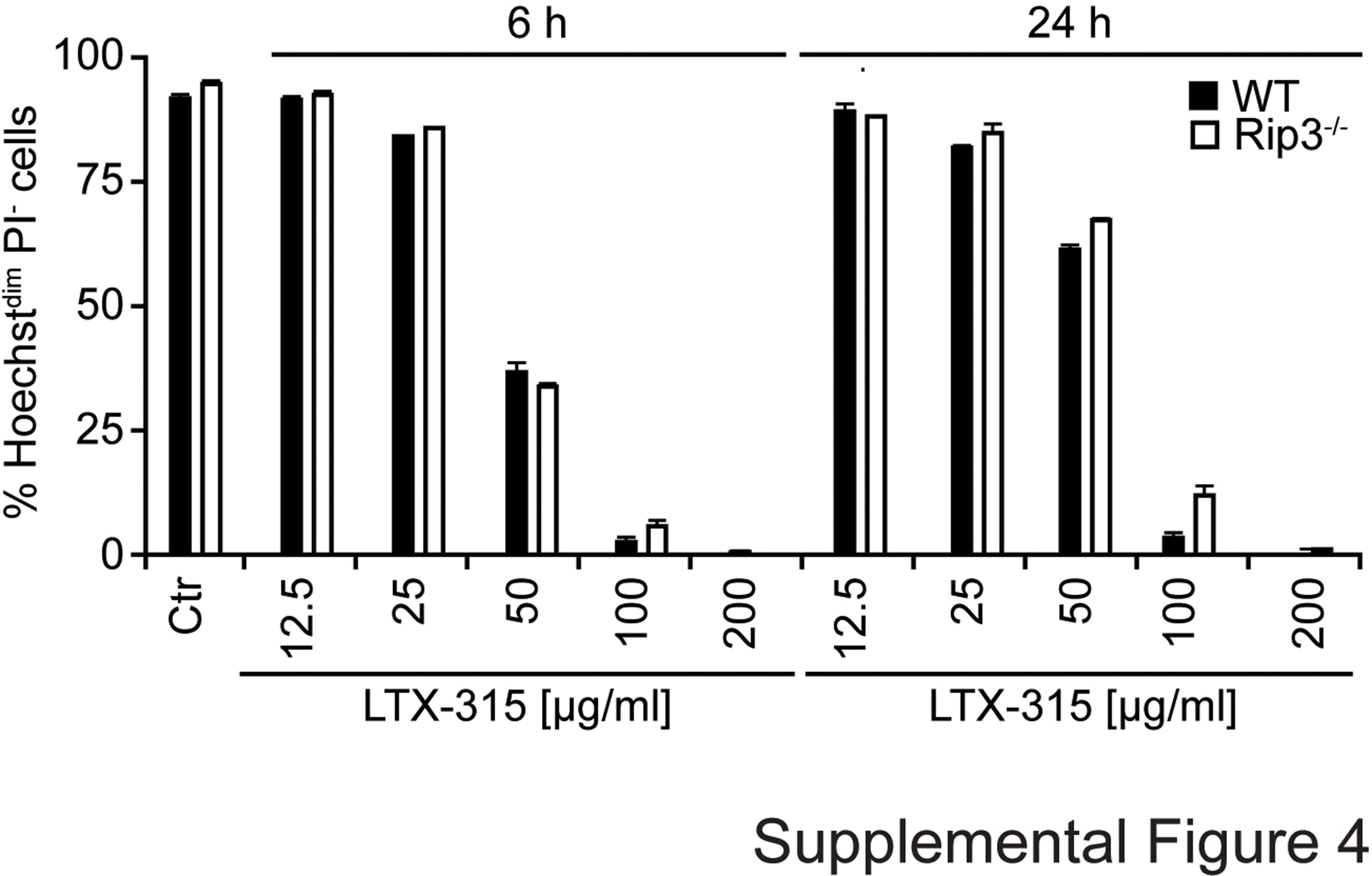

Supplement: Supplementary Figure 4 [file cddis201647x4.tif]

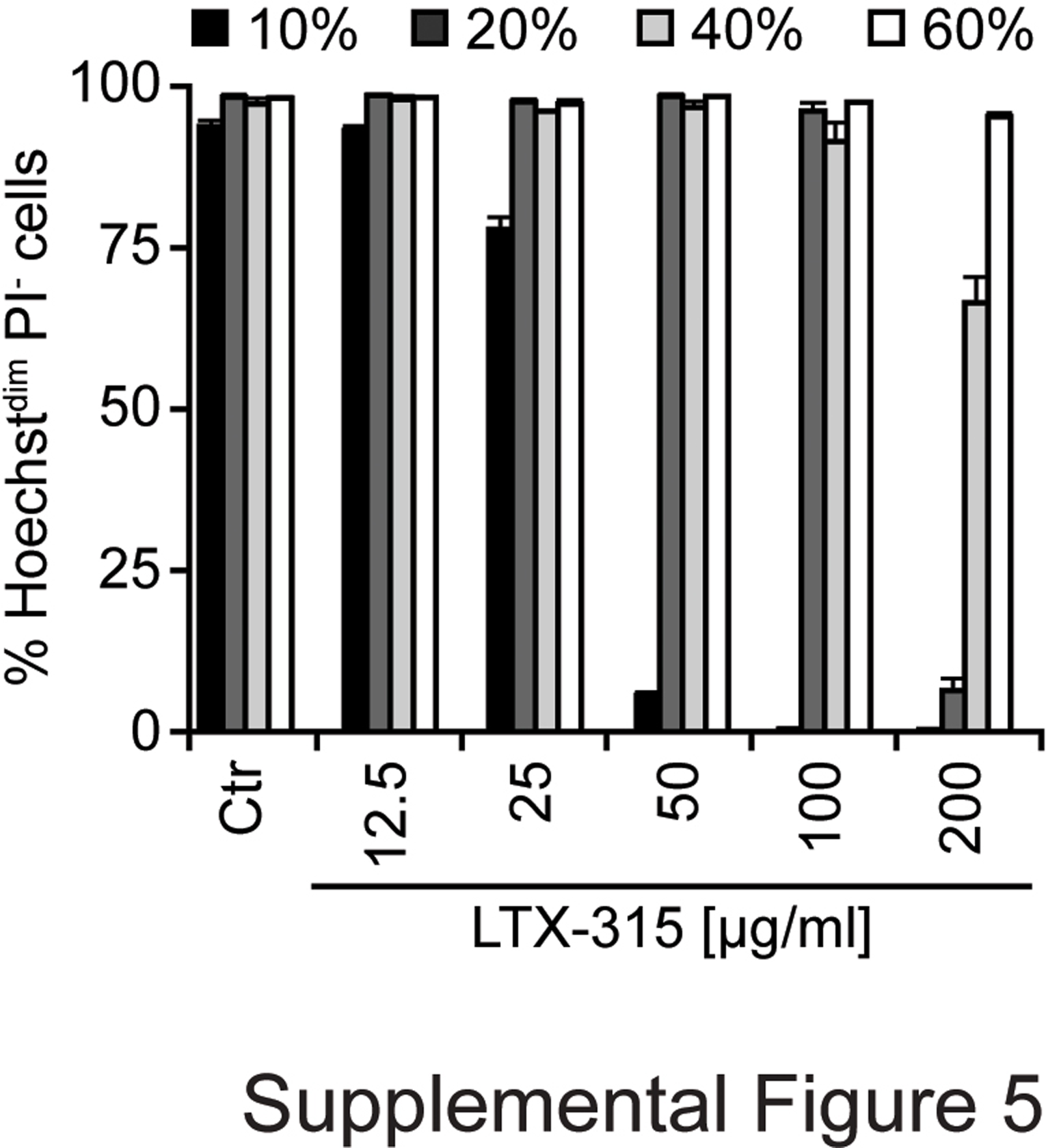

Supplement: Supplementary Figure 5 [file cddis201647x5.tif]
