# Supplementary material for: The oncolytic peptide LTX-315 triggers immunogenic cell death
Source: Cell Death Dis. 2016 Mar 10;7(3):e2134–. doi: 10.1038/cddis.2016.47 (PMC4823948; doi:10.1038/cddis.2016.47)
Supplement: Supplementary Figure Legends [file cddis201647x6.doc]

**SUPPLEMENTAL FIGURE LEGENDS**

**Supplemental Figure 1.** Effects of LTX-315 on cellular viability. U2OS cells were treated for the indicated period (6 or 24 h) with LTX-315, mitoxantrone (MTX) or carbonyl cyanide m-chlorophenyl hydrazine (CCCP). The frequency of adherent cells that exclude PI and have a normal nuclear morphology (measured within 4 view fields per well) was calculated by automatic image analysis. Quantitative results are shown as means ± SD of 3 wells. They correspond to Fig. 1. Asterisks indicate significant differences (unpaired Student t test) with respect to untreated controls. *p<0.05; **p<0.01; ***p<0.001.

**Supplemental Figure 2.** Failure of LTX-315 to induce eIF2α phosphorylation. **A,B.** U2OS cells were treated for the indicated time with the indicated concentrations of LTX-315 or, alternatively with thapsigargin (THAP), the positive control. Then, cells were fixed and permeabilized, followed by immunofluorescence detection of phosphorylated eIF2α. Representative microphotographs are shown in A. Quantitative results (means±SD of triplicates) are shown in B. Asterisks indicate significant differences (unpaired Student *t* test) with respect to untreated controls. *p<0.05; **p<0.01; ***p<0.001.

**Supplemental Figure 3.** HMGB1 release from tumor cell nuclei in response to LTX-315 *in vivo*. Subcutaneous MCA205 fibrosarcomas were injected locally with PBS (control, Ctr) or LTX-315. Four days later, the tumors were retrieved and subjected to immunofluorescence staining to detect HMGB1 and counterstained with Hoechst 33342. Note that in response to LTX-315 a major portion of the tumor is necrotic (dotted lines) and exhibits reduced HMGB1 staining.

**Supplemental Figure 4.** Cell death induced by LTX-315 in RIP3 knockout cells. TC1 cells were treated for the indicated period (6 or 24 h) with LTX-315. The frequency of adherent cells that exclude PI and have a normal nuclear morphology (measured within 4 view fields per well) was calculated by automatic image analysis. Quantitative results are shown as means ± SD of 3 wells.

**Supplemental Figure 5.** Inhibition of LTX-315 by serum. U2OS cells were treated for 24 h with LTX-315 in the presence of the indicated concentration of serum. The frequency of adherent cells that exclude PI and have a normal nuclear morphology (measured within 4 view fields per well) was calculated by automatic image analysis. Quantitative results are shown as means ± SD of 3 wells.
